# Supplementary material for: Utilization of Staphylococcal Immune Evasion Protein Sbi as a Novel Vaccine Adjuvant
Source: Front Immunol. 2019 Jan 11;9:3139. doi: 10.3389/fimmu.2018.03139 (PMC6336717; doi:10.3389/fimmu.2018.03139)
Supplement: Supplementary file 1 [file Data_Sheet_1.pdf]

Supplementary Information for:

Utilisation of staphylococcal immune evasion protein Sbi as a novel vaccine adjuvant

Yang, Y. ^{§1}, Back, CR.^{§1}, Gräwert, MA.², Wahid, AA.¹, Denton, H.³,
Kildani, R.³, Paulin, J.³, Wörner, K.⁴, Kaiser, W.⁴, Svergun, DI.²,
Sartbaeva, A.⁵ Watts, AG.⁶, Marchbank, KJ.^{3*},
and van den Elsen, JMH^{1*}.

¹Department of Biology and Biochemistry, University of Bath, Bath, UK

²European Molecular Biology Laboratory, Hamburg Unit
c/o Deutsches Elektronen-Synchrotron, EMBL Hamburg, Germany

³Institute of Cellular Medicine, Newcastle University, Newcastle-upon-Tyne, UK

⁴Dynamic Biosensors GmbH, Martinsried, Germany

⁵Department of Chemistry, University of Bath, Bath, UK

⁶Department of Pharmacy and Pharmacology, University of Bath, Bath, UK

This PDF file includes:

Supplementary Table S1 and S2

Supplementary Figures S1 – S5

Contents of Supplementary Information, Yang *et al.*:

Supplementary Tables:

**Table S1**	**Mutagenic primers for amino acid substitutions in Sbi-III-IV**
**Table S2**	**SAXS analysis (data acquisition, analysis and software)**

Supplementary Figures:

**Figure S1**	**A) SDS-PAGE profiles of purified Sbi-III-IV domain III amino acid substitution mutants.<br>B) Anti-C3d western blot analysis of human serum treated with Sbi-III-IV single mutants<br>C) Anti-Sbi western blot analysis of human serum treated with Sbi-III-IV single mutants**
**Figure S2**	**A) Kinetic binding analysis of complexes between C3d and Sbi-III-IV mutants<br>B) Hydrodynamic diameter measurements of complexes between C3d and Sbi-III-IV mutants**
**Figure S3**	**A) Surface plasmon resonance evaluation of Sbi-III-IV binding to C3 activation fragments<br>B) Sbi binding subtracted tripartite complexes sensorgrams on C3b surface<br>C) Surface plasmon resonance analysis of tripartite complex formation on an iC3b surface<br>D) Surface plasmon resonance analysis of tripartite complex formation on a C3d surface<br>E) Surface plasmon resonance reconstitution of FHR or tripartite complex bound C3 convertase<br>F) Fluorometric assay of fluid C3b breakdown**
**Figure S4**	**A-C) SAXS analyses of the Sbi-III-IV(K173A):C3d complex**
**Figure S5**	**A) Design of the Sbi-III-IV-Ag85b fusion construct<br>B) Circular Dichroism analysis of the Sbi-III-IV-Ag85b fusion construct<br>C) SAXS analysis of the Sbi-III-IV-Ag85b fusion construct<br>D) Alternative pathway activation analysis of the Sbi-III-IV-Ag85b fusion construct.<br>E) Western blot analysis of C3 activation and C3-fragment deposition in NHS after incubation with Ag85b alone, Ag85b + Sbi-III-IV and Sbi-III-IV-Ag85b fusion construct.**

Mutation	Primer (5'-3')
S213A	F:gtcatgatgagcgtgtgaaa **gca** gcaaatgatgcaatct
	R:agattgcatcattttgc **tgc** tttcacacgctcatcatgac
R231A	F:atgaaaaagattcaattgaaaacaga **gct** tttagcacaacgtgaagttaacaaag
	R:ctttgttaacttcacgttgtgctaa **agc** tctgttttcaattgaatctttttcat
K159A	F:tccgaacgtcaaaaatttgaaaatgcggat **gca** gcaattaaagatttccaag
	R:cttggaatctttaattgc **tgc** atccgcattttcaatattttgacgttcgga
K162A	F:caaaatattgaaaatgcggataaagcaatt **gca** gatttccaagataacaaagcacc
	R:gggtgctttgttatcttggaatc **tgc** aattgctttatccgcattttcaatattttg
K168A	F:cggataaagcaattaaagatttccaagataac **gca** gcaccacacgataaatca
	R:tgatttatcgtgtgg **tgc** tgcgttatcttggaatctttaattgctttatccg
K173A	F:aagataacaaagcaccacacgat **gca** tcagcagcatatgaagctaac
	R:gtttagcttcatatgctgctga **tgc** atcgtgtggtgctttgttatctt
S174A	F:gcttcatatgctgct **gct** ttggcgtgtggtgcttt
	R:aaagcaccacacgcca **agc** agcagcatatgaagc
S199A	F:ataaccgctttgtagaaaaagtt **gca** attgaaaaagcaatcgttcgt
	R:acgaacgattgctttttcaat **tgc** aactttttctacaaagcggttat
K212A	F:gttcgtcatgatgagcgtgtg **gca** tcagcaaatgatgcaatctc
	R:gagattgcatcatttgctga **tgc** acacgctcatcatgacgaac
S226A	F:tgcaatctcaaaattaaatgaaaaagat **gca** attgaaaacagacgttttagcac
	R:gtgctaaacgtctgttttcaat **tgc** atctttttcatttaattttgagattgca
Y177F	F:caccacacgataaatcagcagca **ttt** gaagctaactca
	R:tgagttagcttc **aaa** tgctgctgatttatcgtgtggtg
K182A	F:atcagcagcatatgaagctaactca **gca** ttacctaagatttacgcgataaa
	R:tttatcgcgtaaatcttttaggtaa **tgc** tgagttagcttcatatgctgctgat
K185A	F:cagcagcatatgaagctaactcaaaattacct **gca** gatttacgcgataaaaa
	R:tttttatcgcgtaaatc **tgc** aggtaattttgagttagcttcatatgctgctg
K190A	F:ctcaaaattacctaagatttacgcgat **gca** aataaccgctttgtagaaaaagtttca
	R:tgaaactttttctacaaagcggttatt **tgc** atcgcgtaaatcttttaggtaattttgag

Table S1. Mutagenic primers for single amino acid substitutions in Sbi-III-IV. Mutated codons are highlighted in red.

Table S2. SAXS table (data acquisition, data analysis and software)¹

(a) SAS data collection parameters			
Source, instrument and description or reference	Hamburg, Petra 3		
Wavelength (Å)	1.24		
Beam geometry (size, sample-to-detector distance)	0.2 x 0.12 mm ³ , 3.0 m		
<i>q</i> -measurement range (Å ⁻¹)	0.01-0.5		
Basis for normalization to constant counts	Diode reading of beam transmission (on beam stop)		
Method for monitoring radiation damage, X-ray dose where relevant	Batch mode: comparison of single frames<br>SEC-SAXS: analysis of background fluctuation		
Exposure time, number of exposures	Batch: 20 x 50 msec<br>SEC SAXS 1 sec frames		
Sample temperature (°C)	20°C		
(b) Software employed for SAS data reduction, analysis and interpretation			
SAS data reduction to sample-solvent scattering, and extrapolation, merging, etc:<br><i>SASFlow</i> ²			
Basic analyses: Guinier, <i>P(r)</i> , scattering particle volume (<i>e.g.</i> Porod volume <i>V_P</i> or volume of correlation <i>V_c</i>)<br><i>ATSAS</i> ³ [REF]			
Shape/bead modelling<br><i>DAMMIF</i> (<i>ATSAS</i>) ⁴			
Atomic structure modelling (homology, rigid body, ensemble)<br><i>EOM</i> (<i>ATSAS</i>) ⁵			
Modelling of missing sequence from PDB files:<br><i>CORAL</i> (<i>ATSAS</i>) ⁶			
Molecular graphics<br><i>The PyMOL Molecular Graphics System, Version 2.0, Schrödinger, LLC.</i>			
(c) Structural parameters			
Guinier Analysis	Sbi-III-IV:C3d:FHR1	Sbi-III-IV(K173A):C3d	Sbi-III-IV-Ag85b
<i>I</i> (0) (cm ⁻¹)	0.033 +/- 0.0002	0.024 +/- 0.0005	0.016 +/- 0.0002
<i>R_g</i> (Å)	83.2 +/- 0.4	30.6 +/- 0.2	36.6 +/- 0.3
<i>q</i> -range (Å ⁻¹)	0.01 – 0.2	0.01 – 0.5	0.01 – 0.5
<i>P(r)</i> analysis			
<i>I</i> (0) (cm ⁻¹)	0.033	0.024 +/- 0.0005	0.016 +/- 0.0002
<i>R_g</i> (Å)	83.8 +/- 0.5	30.6 +/- 0.2	36.7 +/- 0.2
<i>d_{max}</i> (Å)	303	108	135
<i>q</i> -range (Å ⁻¹)	0.01 – 0.1	0.01 – 0.26	0.01 – 0.22

¹ Sample details are listed in Material & Method section² Franke, D., Kikhney, A.G. and Svergun, D.I. (2012) Automated acquisition and analysis of small angle X-ray scattering data. *Nuc. Inst. Meth. A.* 689, 52-59³ Franke, D., Petoukhov, M.V., Konarev, P.V., Panjkovich, A., Tuukkanen, A., Mertens, H.D.T., Kikhney, A.G., Hajizadeh, N.R., Franklin, J.M., Jeffries, C.M. and Svergun, D.I. (2017) *ATSAS 2.8: a comprehensive data analysis suite for small-angle scattering from macromolecular solutions.* J. Appl. Cryst. 50⁴ Franke, D. and Svergun, D.I. (2009) *DAMMIF*, a program for rapid ab-initio shape determination in small-angle scattering. J. Appl. Cryst., 42, 342-346.⁵ Tria, G., Mertens, H. D. T., Kachala, M. & Svergun, D. I. (2015) Advanced ensemble modelling of flexible macromolecules using X-ray solution scattering. *IUCr* 2, 207-21⁶ Petoukhov, M.V., Franke, D., Shkumatov, A.V., Tria, G., Kikhney, A.G., Gajda, M., Gorba, C., Mertens, H.D.T., Konarev, P.V. and Svergun, D.I. (2012) New developments in the *ATSAS* program package for small-angle scattering data analysis. J. Appl. Cryst. 45, 342-350

Quality-of-fit parameter (GNOM ⁷)	0.67	0.51	0.58
Porod Volume (nm ³)	406	80.5	66.2
M from Porod	238 kD	47.3 kD	40kD
M from I(0) (ratio to expected value)	210 kD (1.2)	56 kD (1.2)	n.A. SEC-SAXS (SLS = 53 kD (1.06))

(d) Shape modelling results (a complete panel for each method)

	Sbi-III-IV:C3d:FHR1	Sbi-III-IV(K173A):C3d	Sbi-III-IV-Ag85b
<i>q</i> -range for fitting	na	Dammif	n.A.
Symmetry/anisotropy assumptions	na	P1	P1
Model volume and/or <i>M</i> estimate	na	46 kD	n.A.
Model precision/resolution	na	29 +/-28	n.A.

(e) Atomistic modelling

	Sbi-III-IV:C3d:FHR1	Sbi-III-IV(K173A):C3d	Sbi-III-IV-Ag85b
Method	EOM	CORAL (missing Atoms)	EOM
<i>q</i> -range for fitting	0.01-0.2	0.01-0.25	0.01-0.25.
Symmetry assumptions	P2	P1	P1.
χ^2 value/range	3.2	1.8-2.9	1.2
Relevant output parameters (predicted R_g/d_{max} values for multi-state models)	73.75/238.51	n.a.	n.a.
Domain/subunit coordinates and contacts, regions of presumed flexibility as appropriate	see material and methods		

(g) Data and model deposition IDs

	Sbi-III-IV:C3d:FHR1	Sbi-III-IV(K173A):C3d	Sample 3 <i>etc.</i>
**Not yet applicable, will be added**			

⁷ Svergun D.I. (1992) Determination of the regularization parameter in indirect-transform methods using perceptual criteria. *J. Appl. Crystallogr.* **25**, 495-503

⁸ A.T. Tuukkanen, G.J. Kleywegt and D.I. Svergun (2016) Resolution of ab initio shapes determined from small-angle scattering IUCrJ **3**, 440-447.

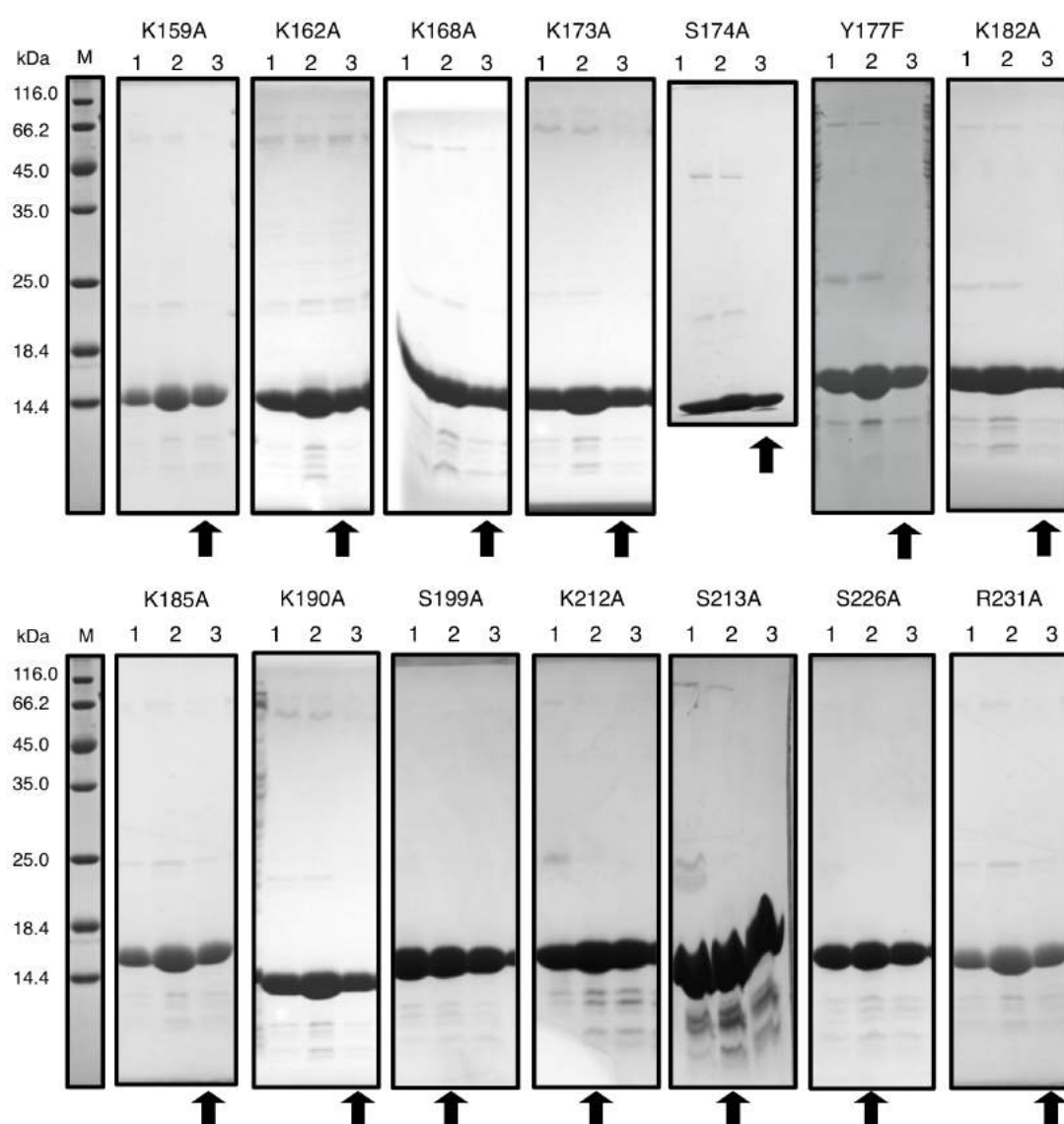

Figure S1A. SDS-PAGE profiles of purified Sbi-III-IV single amino acid substitution mutants. Each mutant was purified by Ni^{2+} affinity chromatography with 1 ml Histrap FF column. Three peak fractions (lanes 1-3) were analyzed by SDS-PAGE and shown as a single gel segment. All Sbi-III-IV mutants display the expected size of 14.9 kDa, located between 18 kDa and 14.4 kDa molecular weight marker. The purest fraction of each purification was used for subsequent studies (indicated by black arrows).

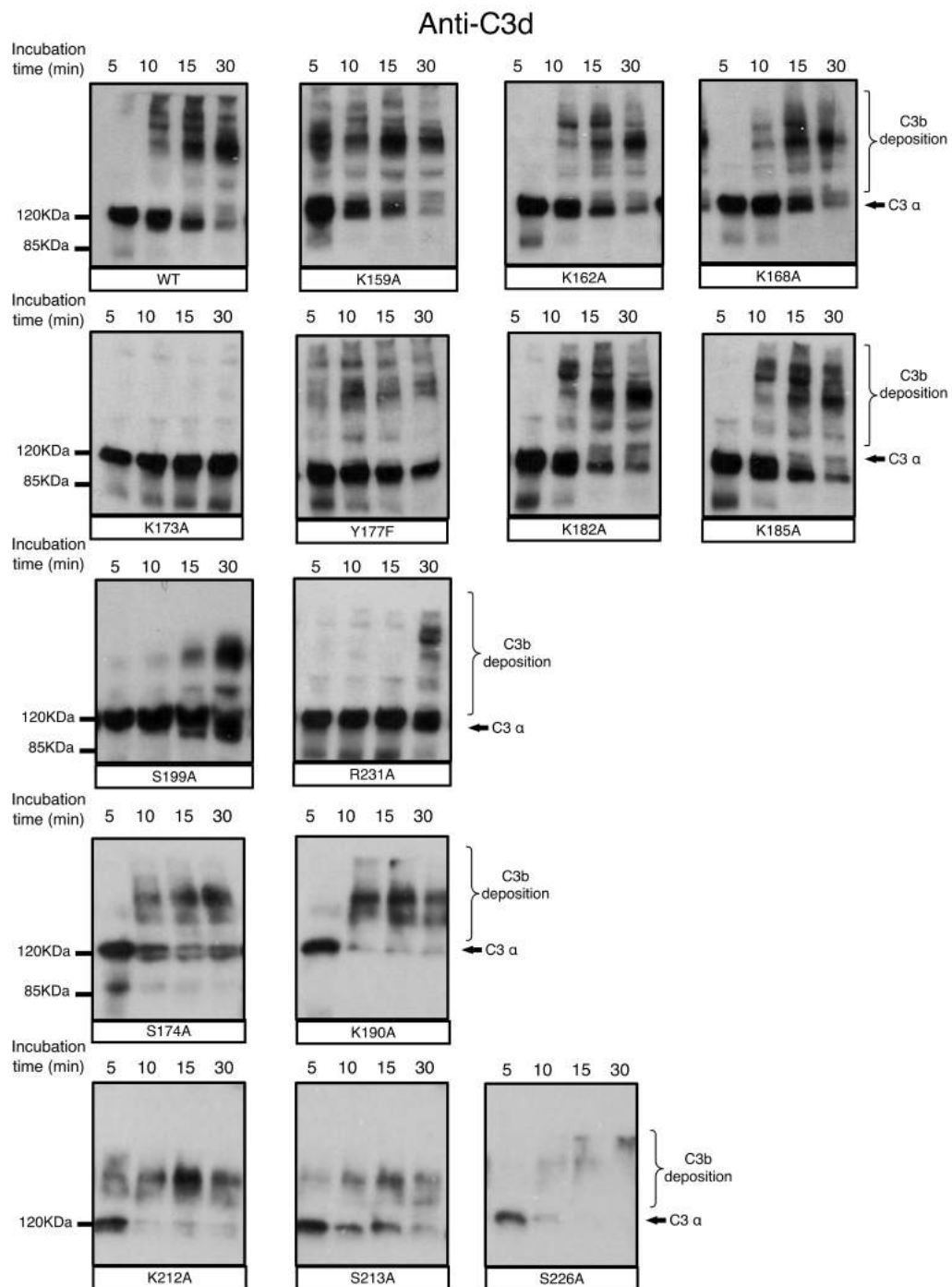

Figure S1B. Anti-C3d western blot analysis of human serum treated with Sbi-III-IV single mutants.

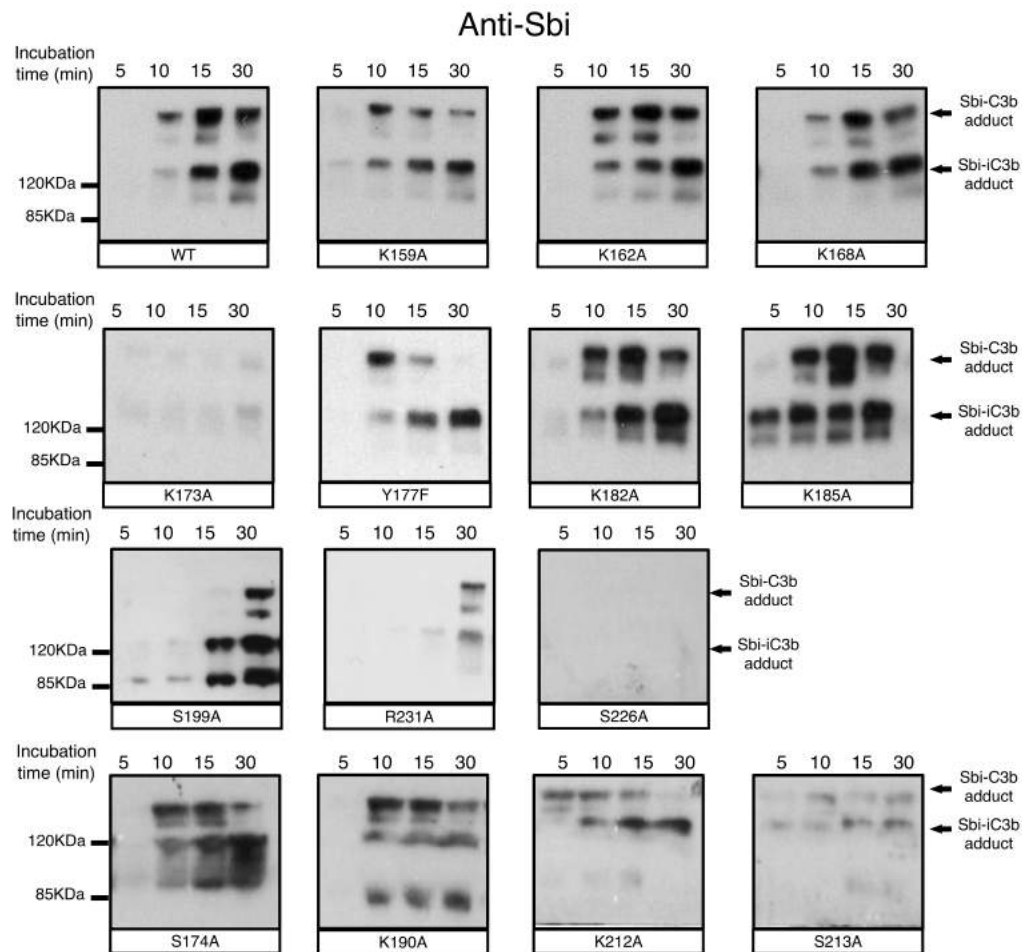

Figure S1C. Anti-Sbi western blot analysis of human serum treated with Sbi-III-IV single mutants.

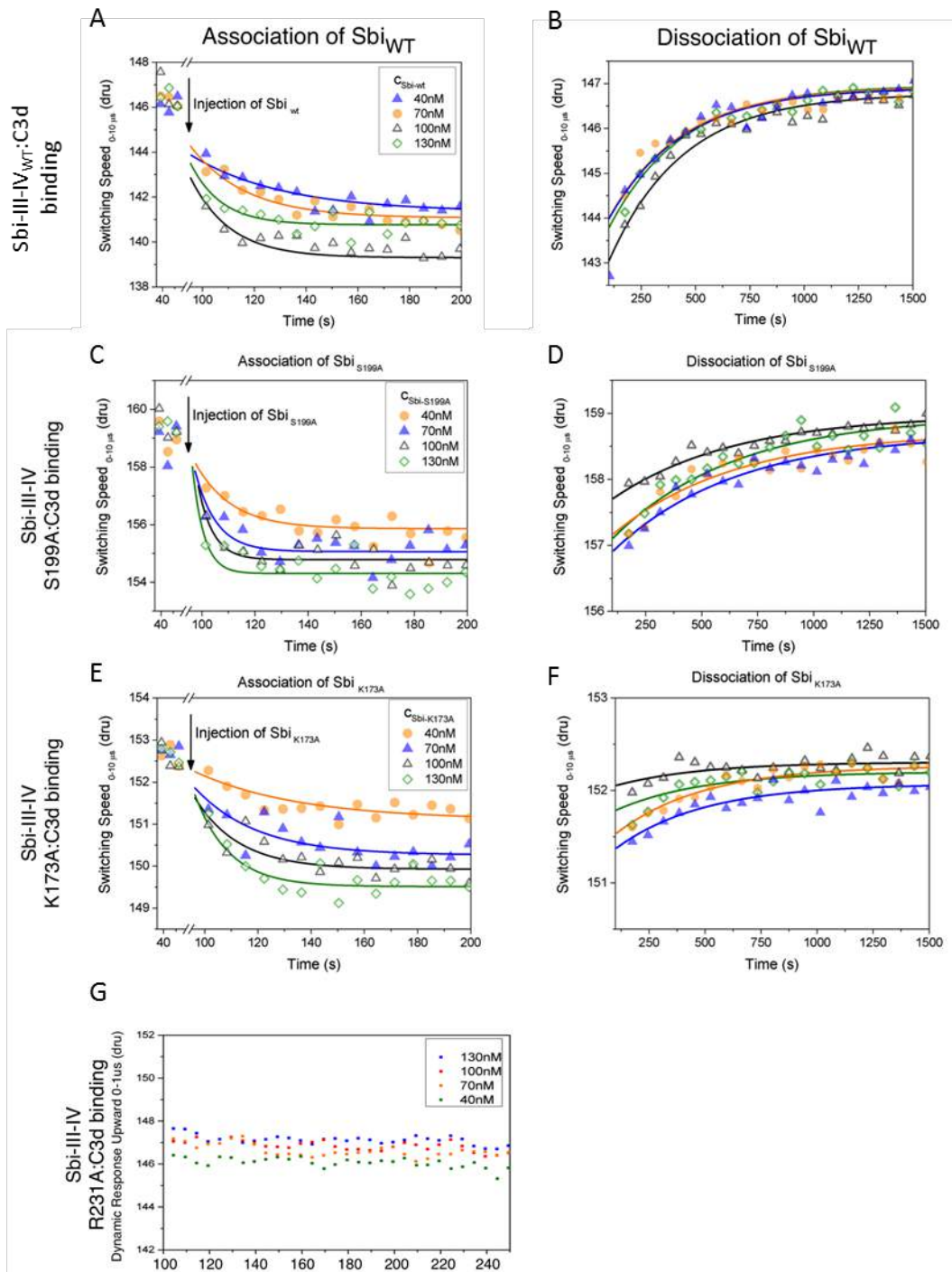

Figure S2A. Kinetic binding analysis of complexes between C3d and Sbi-III-IV mutants. (A-B) Time resolved dynamic response of immobilized Sbi-III-IV WT during C3d association and dissociation. (C-D) Time resolved dynamic response of immobilized Sbi-III-IV S199A during C3d association and dissociation. (E-F) Time resolved dynamic response of immobilized Sbi-III-IV K173A during C3d association and dissociation. (G) Time resolved dynamic response of immobilized Sbi-III-IV R231A during C3d association. Upward dynamic response readings were recorded every 5s. The association and dissociation curves were determined by global fitting and used to calculate the kinetic parameters.

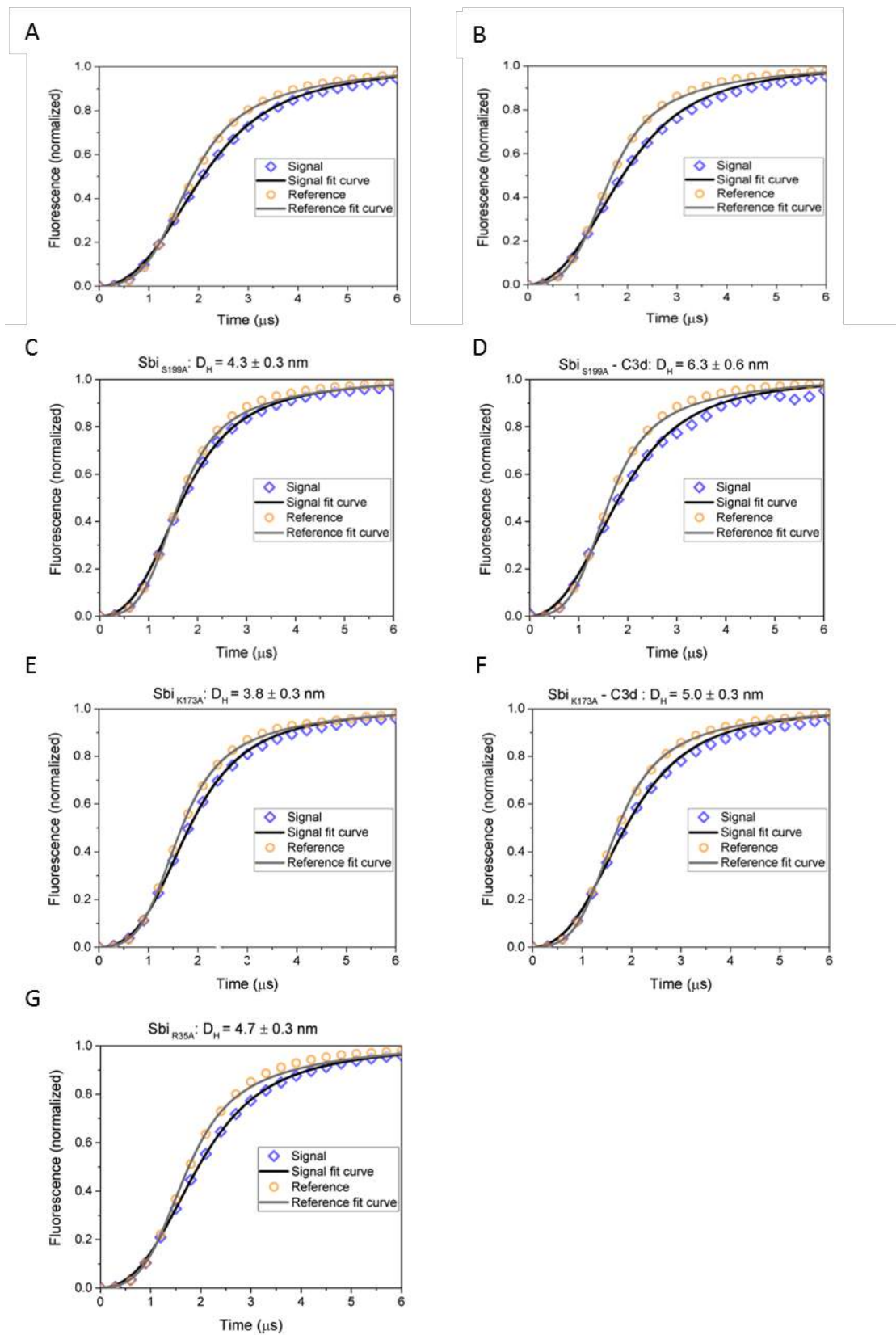

Figure S2B. Hydrodynamic diameter measurements of complexes between C3d and Sbi-III-IV mutants. (A) Sbi-III-IV WT. (B) Sbi-III-IV:C3d complex. (C) Sbi-III-IV S199A mutant. (D) Sbi-III-IV_{S199A}:C3d complex. (E) Sbi-III-IV K173A mutant. (F) Sbi-III-IV_{K173A}:C3d complex. (G) Sbi-III-IV R231A mutant.

A

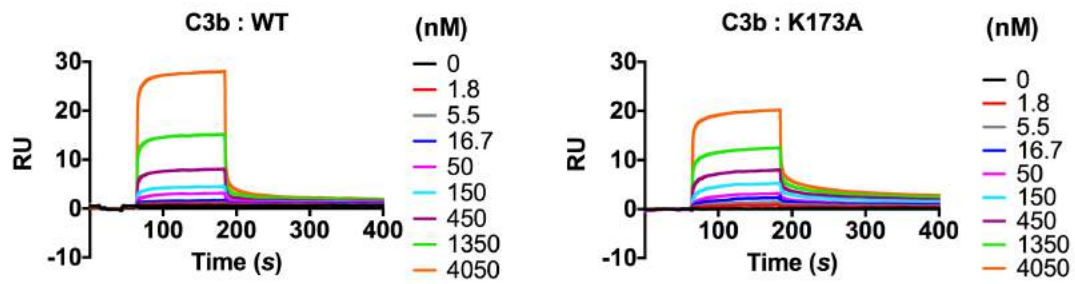

B

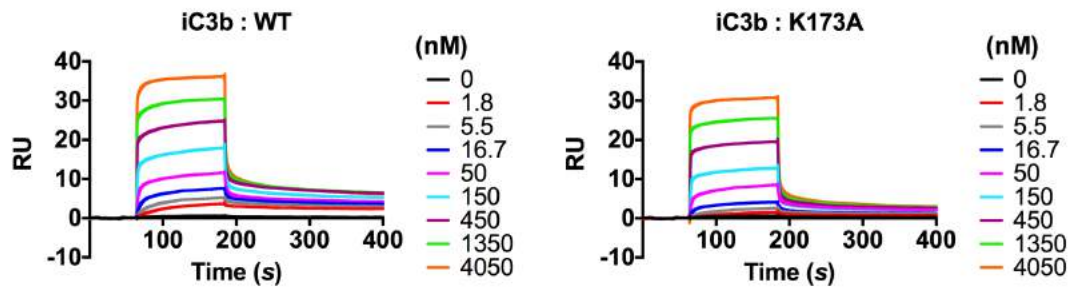

C

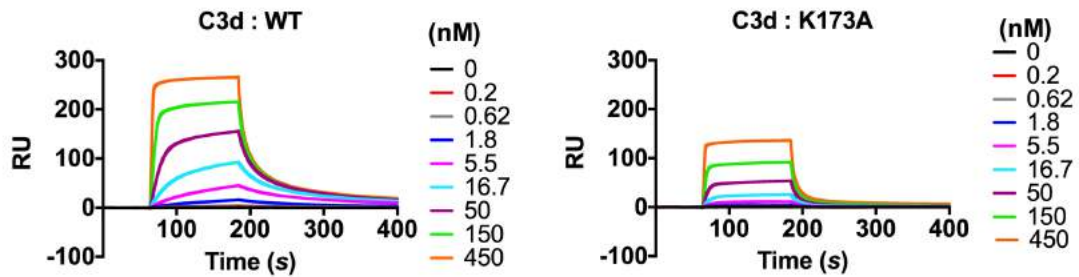

Figure S3A. Surface plasmon resonance evaluation of Sbi-III-IV binding to C3 activation fragments. (A) On an opsonised C3b surface, a triply diluted concentration series (4050 to 1.8 nM) of either WT or K173A Sbi-III-IV was flowed across. (B) The same experiment procedure was performed on a opsonised iC3b surface. (C) On an amine coupled C3d surface, a triply diluted concentration series (450 to 0.2 nM) of either WT or K173A Sbi-III-IV was flowed across. Each sensorgram is representative of two experiments.

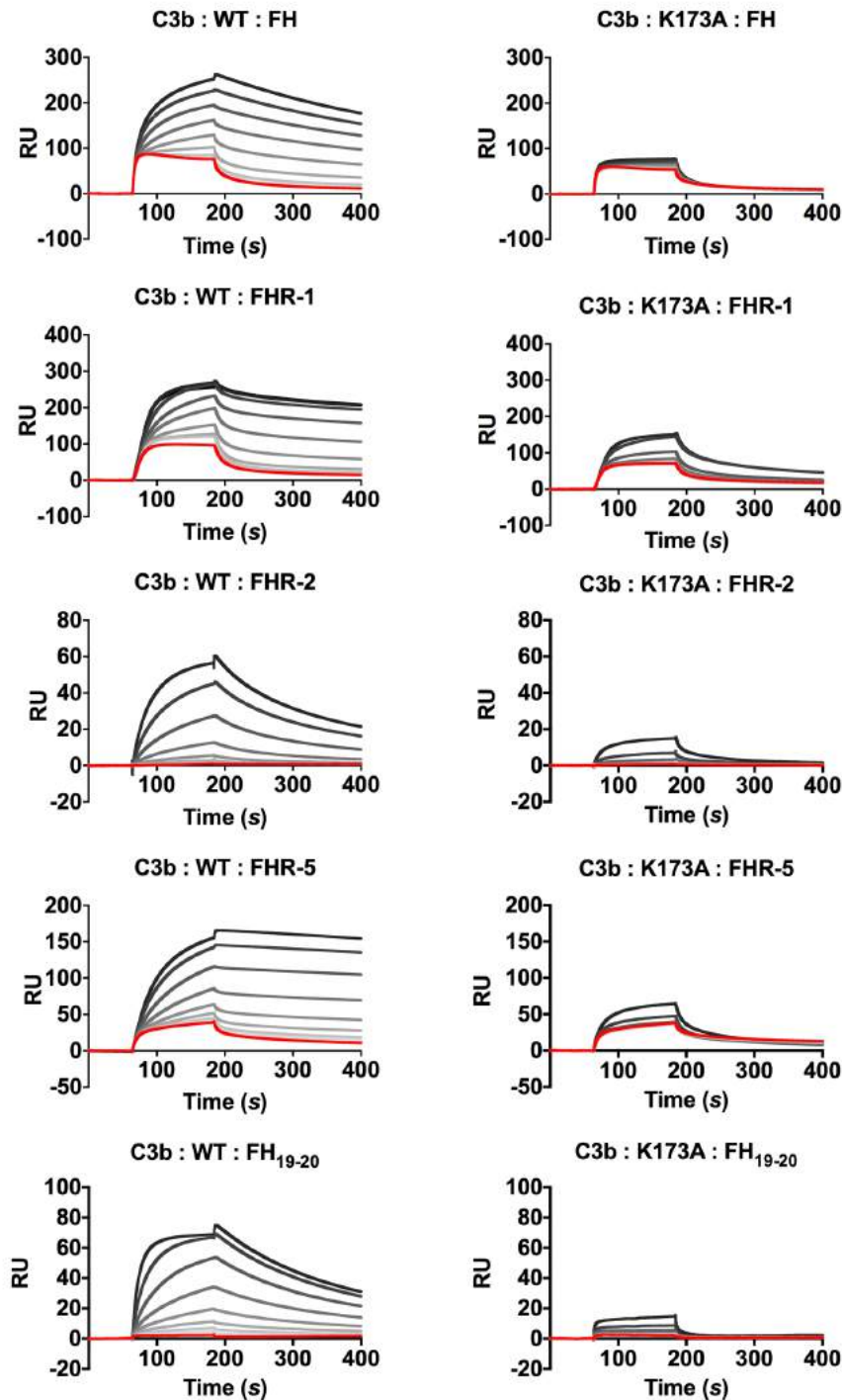

Figure S3B. Sbi binding subtracted tripartite complexes sensorgrams on C3b surface. The figures were generated by subtracting the co-injection sensorgrams (Figure 2) with the corresponding Sbi binding dataset (Supplementary Figure S3AA). The red response curves were indicative of binding experiment in the absence of Sbi. The co-injection experiments of a fixed analyte concentration in combination with increasing Sbi concentration (1.8 to 4050 nM) were depicted by increasingly dark lines. WT, Wild type Sbi-III-IV. K173A, a point substitution mutant of Sbi-III-IV.

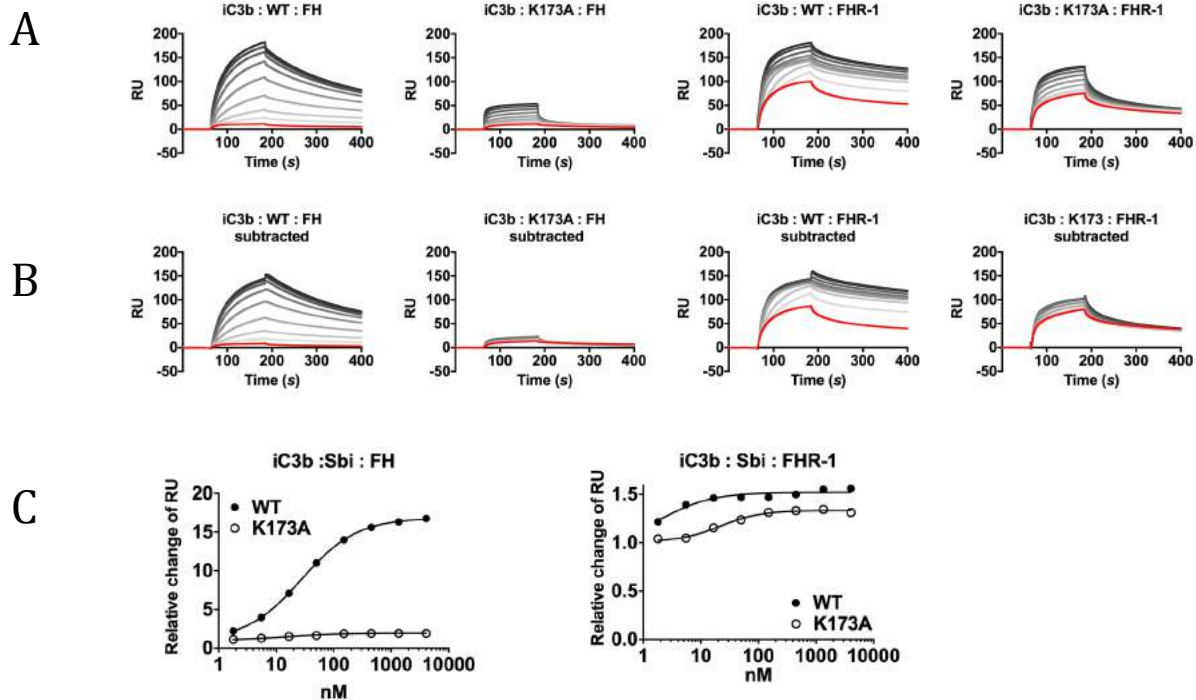

Figure S3C. Surface plasmon resonance analysis of tripartite complex formation on an iC3b surface. (A) A triply diluted concentration series of either WT or K173A Sbi-III-IV (4050 to 1.8 nM) was co-injected with plasma purified FH (100 nM) or recombinant FHR-1 (12.5 nM). (B) This row of figures were generated by subtracting the co-injection sensorgrams (shown in A) with the corresponding Sbi binding dataset (Supplementary Figure S3AB). The red response curves were indicative of binding experiment in the absence of Sbi. The co-injection experiments of a fixed analyte concentration in combination with increasing Sbi concentration were depicted by increasingly dark lines. (C) Based on Supplementary Figure S3CB, changes in FH (or FHRs) binding were expressed as the relative change, derived from dividing the Sbi mediated binding by the FH (or FHR) only control, using the response-difference values at the equilibrated binding point (173.5 s). Each sensorgram is representative of two experiments. For C, relative change curves were fitted using non-linear variable slope (four parameters) function in GraphPad Prism.

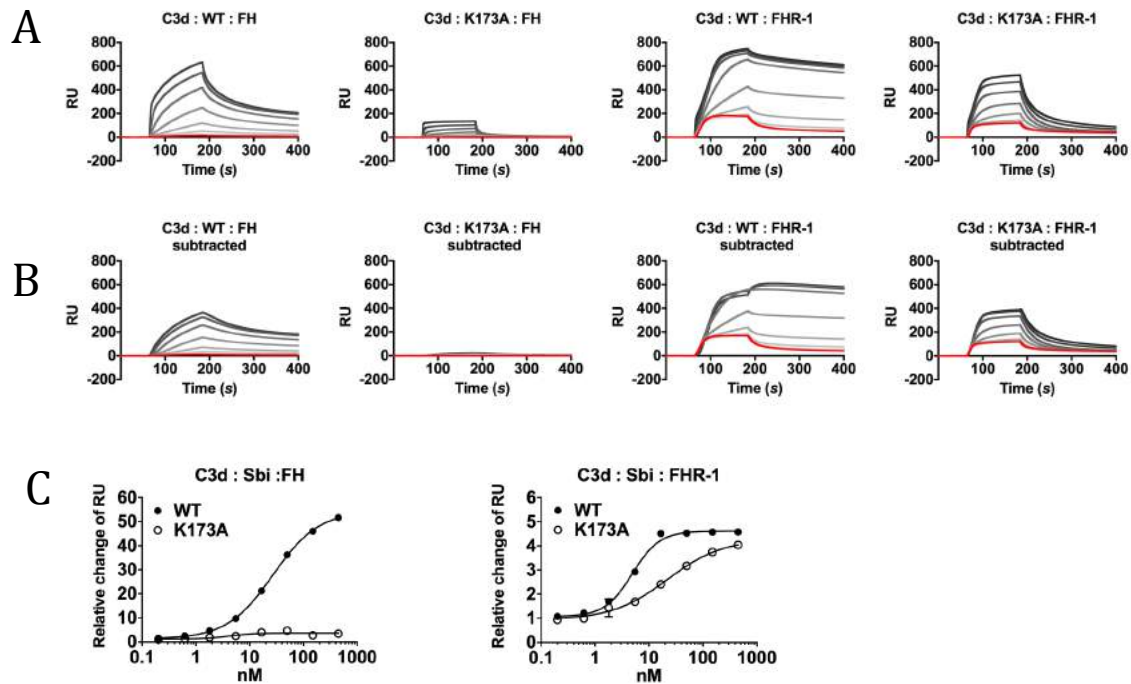

Figure S3D. Surface plasmon resonance analysis of tripartite complex formation on a C3d surface. (A) A triply diluted concentration series of either WT or K173A Sbi-III-IV (450 to 0.2 nM) was co-injected with plasma purified FH (100 nM) or recombinant FHR-1 (12.5 nM). (B) This row of figures were generated by subtracting the co-injection sensorgrams (shown in A) with the corresponding Sbi binding dataset (Supplementary Figure S3AC). The red response curves were indicative of binding experiment in the absence of Sbi. The co-injection experiments of a fixed analyte concentration in combination with increasing Sbi concentration were depicted by increasingly dark lines. (C) Based on Supplementary Figure S3DB, changes in FH (or FHRs) binding were expressed as the relative change, derived from dividing the Sbi mediated binding by the FH (or FHR) only control, using the response-difference values at the equilibrated binding point (173.5 s). Each sensorgram is representative of two experiments. For C, relative change curves were fitted using non-linear variable slope (four parameters) function in GraphPad Prism.

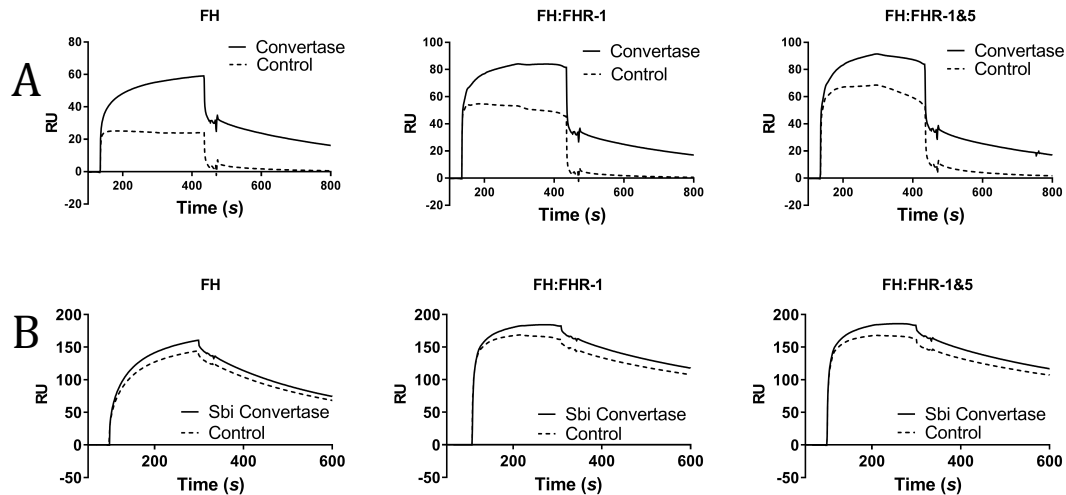

Figure S3E. Surface plasmon resonance reconstitution of FHR or tripartite complex bound C3 convertase. (A) FH (2000 nM) or FH +FHR-1 (2000 and 200 nM) or FH+FHR-1&5 (2000, 200 and 20 nM) was separately flowed across a C3b coated surface in the presence (Solid line, Convertase) or absence (Dashed line, Control) of C3 convertase components (FB+FD). (B) Sbi-III-IV (2 μ M) spiked FH or FH +FHR-1 or FH+FHR-1&5 mixture was separately flowed across a C3b coated surface in the presence (Solid line, Sbi Convertase) or absence (Dashed line, Control) of C3 convertase components (FB+FD). Figure 3g-h were derived by subtracting the control signal from the convertase sensorgram for each condition respectively. Each sensorgram is representative of two experiments.

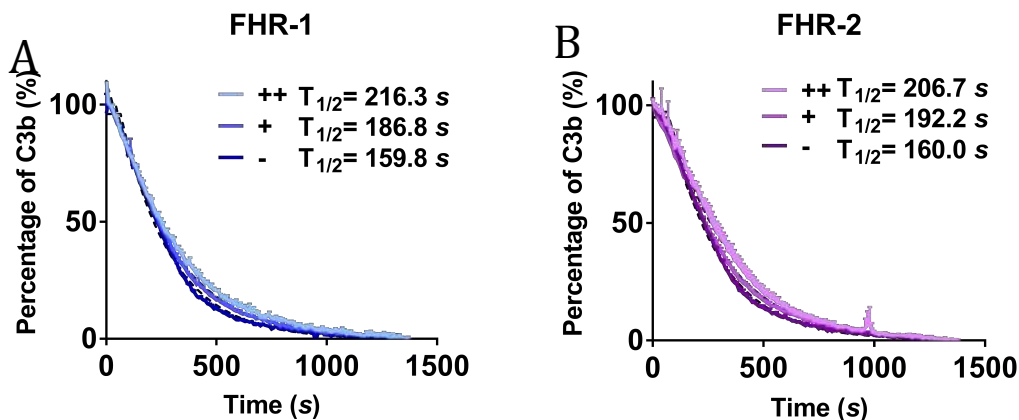

Figure S3F. Fluorometric assay of fluid C3b breakdown. Percentage of intact C3b derived from continuous recording of ANS fluorescence changes between 465-475 nm spectrum. Baseline C3b breakdown curve (-) was recorded in the presence of FH and FI, interference caused by the addition of FHR (+) (FHR-1 (A) and FHR-2 (B)) or both FHRs in combination of Sbi (++) was also examined. Normalized data was depicted in solid lines, simulated breakdown curves were shown as dotted-lines. Each graph is representative of three independent experiments. Simulated breakdown curves were fitted using one phase exponential decay function in GraphPad Prism.

A

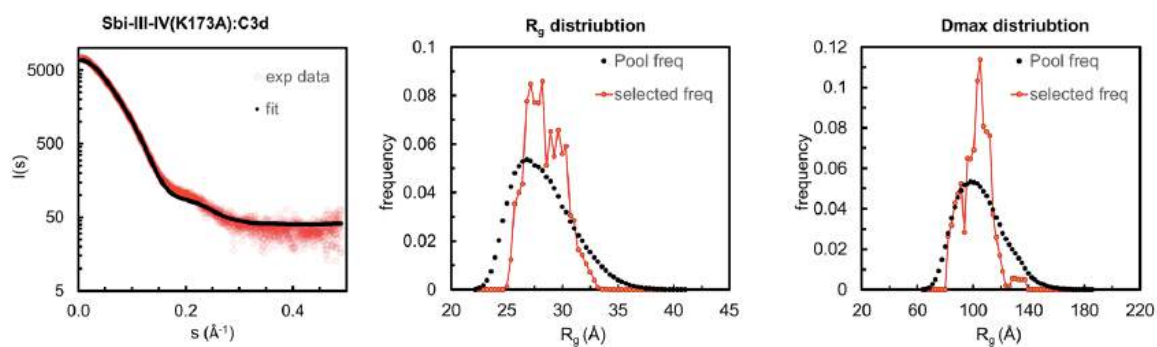

B

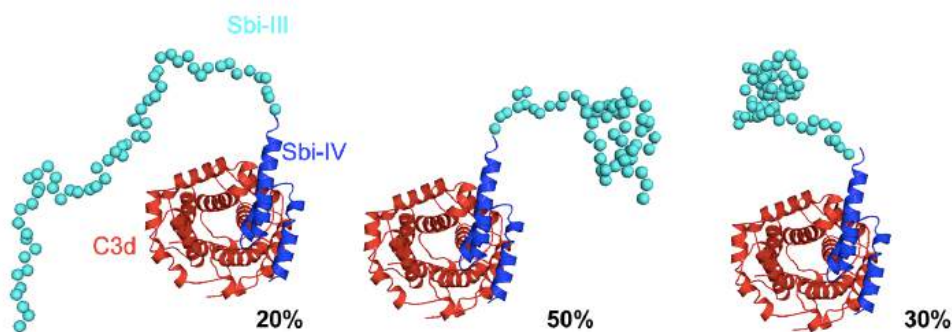

Figure S4A. SAXS analysis of Sbi-III-IV(K173A):C3d complex.

EOM modeling of the Sbi-III-IV(K173A):C3d complex: A) Left panel, fit of the selected ensemble of conformers to the experimental scattering ($\chi^2=3.0$). Middle panel, radius of gyration (R_g) and right panel, maximum dimension of the particle (D_{max}). Distribution histograms of the selected conformers versus the pool are also shown. B) Examples of rigid body models of the selected conformers corresponding to the histogram peaks. The volume fraction of each species is indicated. The relative positions of C3d and Sbi-III-IV are indicated, with C3d in red, Sbi-IV in dark blue and Sbi in turquoise.

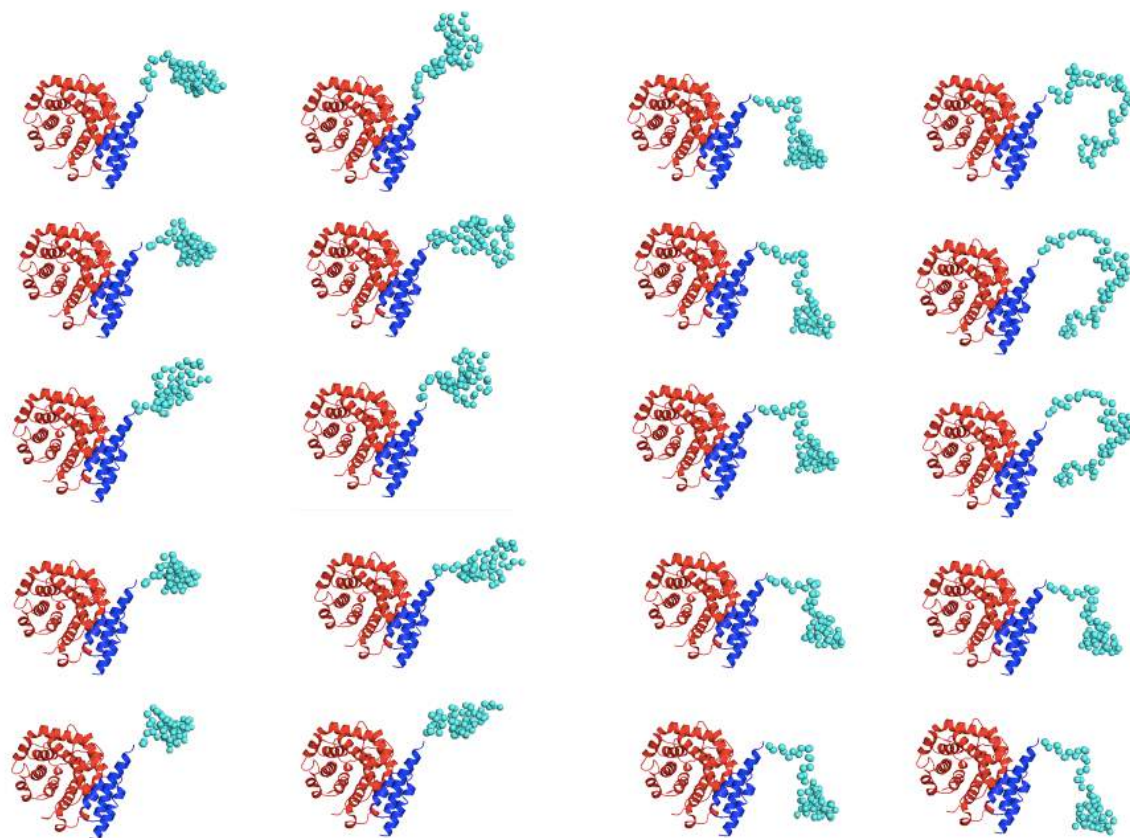

Figure S4B. SAXS analysis of Sbi-III-IV(K173A):C3d complex.

Combined rigid body and *ab initio* modeling approach (CORAL) in part based on the crystal structure of the Sbi-IV:C3d complex (2wy8). The additional 60 N-terminal residues of Sbi-III are included as a connected bead model. C3d is shown in red, Sbi-IV in dark blue and Sbi-III in turquoise.

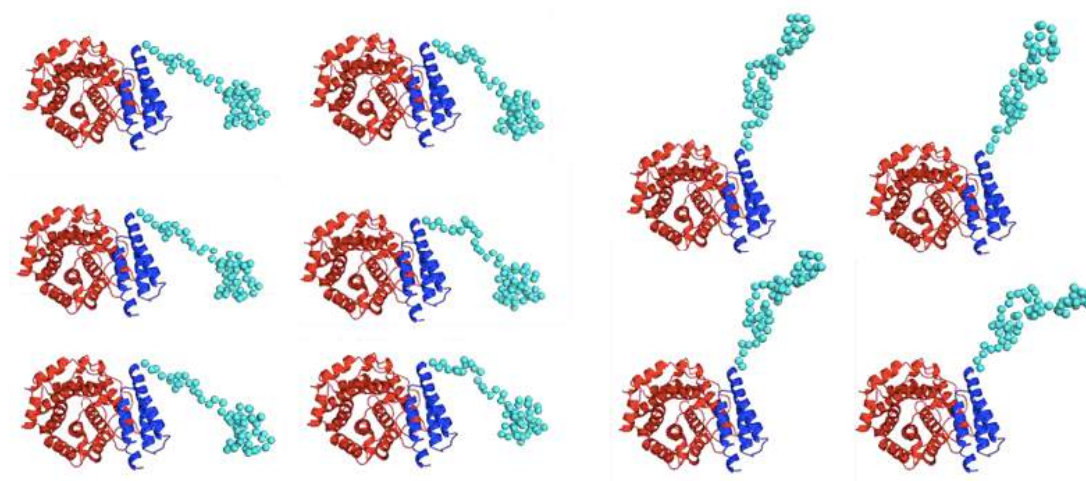

Figure S4C. SAXS analysis of Sbi-III-IV(K173A):C3d complex, alternative binding mode. As described for Supplementary Figure S4A, combined rigid body and *ab initio* modeling approach (CORAL) was performed based on the alternative Sbi-IV:C3d binding mode (2xy7.pdb). In the modeling of this complex improved χ^2 values were achieved ($\chi^2=1.8$ compared to 2.8). A similar restricted flexibility of Sbi-III was observed with this modeling strategy. C3d is shown in red, Sbi-IV in dark blue and Sbi-III in turquoise.

A

1	<u>GGATCC</u> GGAA	CGTCAAAATA	TTGAAAATGC	GGATAAAGCA	ATTAAAGATT
51	TCCAAGATAA	CAAAGCACCA	CACGATAAAT	CAGCAGCATA	TGAAGCTAAC
101	TCAAAATTAC	CTAAAGATTT	ACGCGATAAA	AATAACCGCT	TTGTAGAAAA
151	AGTTTCAATT	GAAAAAGCAA	TCGTTTCGTCA	TGATGAGCGT	GTGAAATCAG
201	CAAATGATGC	AATCTCAAAA	TTAAATGAAA	AAGATTCAAT	TGAAAACAGA
251	CGTTTAGCAC	AACGTGAAGT	TAACAAAGCA	CCTATGGATG	TAAAAGAGCA
301	TTTACAGAAA	CAATTAGACG	CATTAGTAGC	TCAAAAAGAT	GCTGAAAAGA
351	AAGTGGCGGG	TACCTCAGGC	GGAGGTGGAT	CTCATCACCA	TCATCACCAT
401	CACCATCATC	ACTCTGGCGG	AGGTGGATCT	ACTAGTGCTA	GCTTTAGCCG
451	CCCGGGCCTG	CCGGTGGAAAT	ATCTGCAGGT	GCCGAGCCCG	AGCATGGGCC
501	GCGATATTAA	AGTGCAGTTT	CAGAGCGGCG	GCAACAACAG	CCCGGCGGTG
551	TATCTGCTGG	ATGGCCTGCG	CGCGCAGGAT	GATTATAACG	GCTGGGATAT
601	TAACACCCCG	GCGTTTGAAT	GGTATTATCA	GAGCGGCCTG	AGCATTGTGA
651	TGCCGGTGGG	CGGCCAGAGC	AGCTTTTATA	GCGATTGGTA	TAGCCCGGCG
701	TGCGGCAAAG	CGGGCTGCCA	GACCTATAAA	TGGGAAACCT	TTCTGACCAG
751	CGAACTGCCG	CAGTGGCTGT	CCGCGAACCG	CGCGGTGAAA	CCGACCGGCA
801	GCGCGGCGAT	TGGCCTGAGC	ATGGCGGGCA	GCAGCGCGAT	GATTCTGGCG
851	GCGTATCATC	CGCAGCAGTT	TATTTATGCG	GGCAGCCTGA	GCGCGCTGCT
901	GGATCGGAGC	CAGGGCATGG	GCCCGAGCCT	GATTGGCCTG	GCGATGGGCG
951	ATGCGGGCGG	CTATAAAGCG	GCGGATATGT	GGGGCCCGAG	CAGCGATCCG
1001	GCGTGGGAAC	GCAACGATCC	GACCCAGCAG	ATTCCGAAAC	TGGTGGCGAA
1051	CAACACCCGC	CTGTGGGTGT	ATTGCGGCAA	CGGCACCCCG	AACGAACTGG
1101	GCGGCGCGAA	CATTCCGGCG	GAATTTCTGG	AAAACTTTGT	GCGCAGCAGC
1151	AACCTGAAAT	TTCAGGATGC	GTATAACGCG	GCGGGCGGCC	ATAACGCGGT
1201	GTTTAACTTT	CCGCCGAACG	GCACCCATAG	CTGGGAATAT	TGGGGCGCGC
1251	AGCTGAACGC	GATGAAAGGC	GATCTGCAGA	GCAGCCTGGG	CGCGGGC <u>GCT</u>
1301	<u>GAGC</u>				

B

1	MPLLLLLPLL	WAGALAMDVD	ERQNIENADK	AIKDFQDNKA	PHDKSAAYEA	NSKLPKDLRD
61	KNNRFVEKVS	IEKAIVRHDE	RVKSANDAIS	KLNEKDSIEN	RRLAQREVNK	APMDVKEHLQ
121	KQLDALVAQK	DAEKKVAGTS	GGGGSHHHHH	HHHHHSGGGG	STSASFSRPG	LPVEYLQVPS
181	PSMGRDIKVQ	FQSGGNNSPA	VYLLDGLRAQ	DDYNGWDINT	PAFEWYYQSG	LSIVMPVGGQ
241	SSFYSDWYSP	ACGKAGCQTY	KWETFLTSEL	PQWLSANRAV	KPTGSAAIGL	SMAGSSAMIL
301	AAYHPQQFIY	AGSLSALLDR	SQGMGPSLIG	LAMGDAGGYK	AADMWGPSSD	PAWERNDPTQ
361	QIPKLVANNT	RLWVYCGNGT	PNELGGANIP	AEFLENFVRS	SNLKFQDAYN	AAGGHNAVFN
421	FPPNGTHSWE	YWGAQLNAMK	GDLQSSLGAG	KP		

Figure S5A. Design of the Sbi-III-IV-Ag85b fusion protein. (A) DNA sequence of *sbi-III-IV-Ag85b* for insertion into pET15b. Restriction sites are underlined: 5' = BamH1, 3' = Bpu1102I. (B) Final sequence of recombinant Sbi-III-IV-Ag85b protein. Blue = signal sequence, yellow = Sbi-III-IV, green = Ag85b.

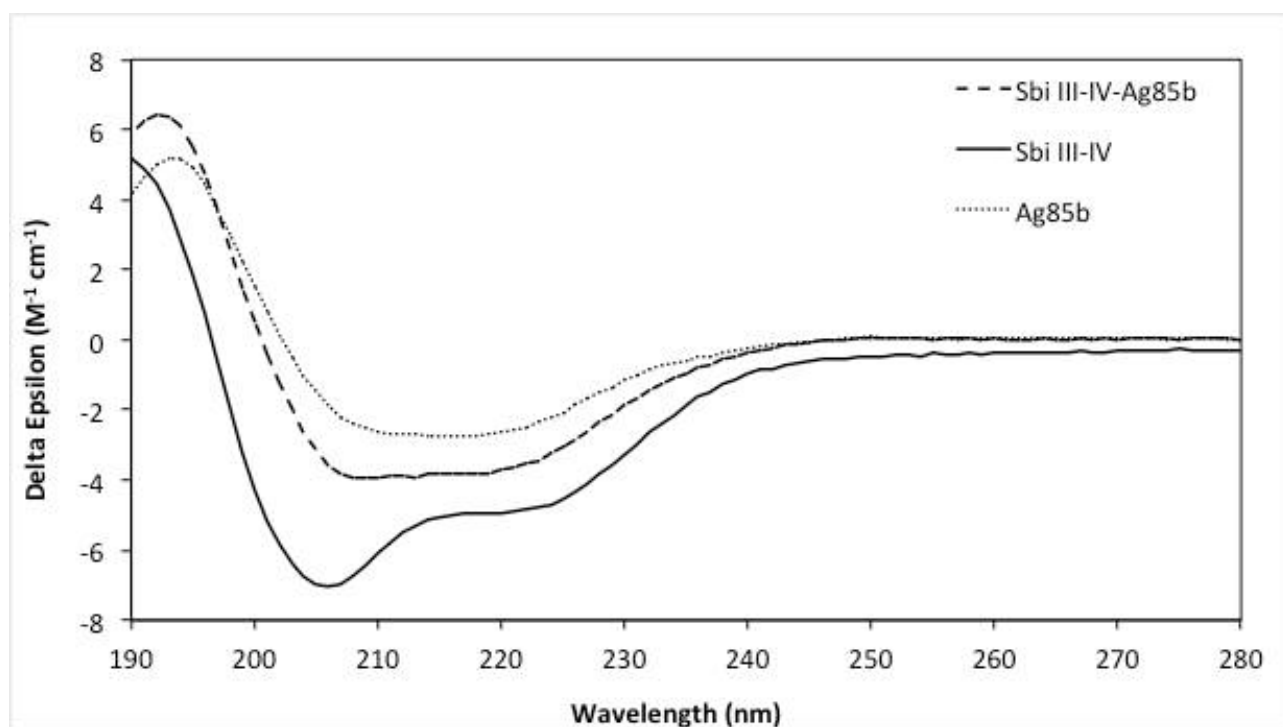

	Reference set for CDSSTR	α helices	β sheets	Turns	Unordered	Total	NRMSD
Sbi III-IV-Ag85b	4	26%	24%	20%	30%	100%	0.015
Sbi III-IV	7	51%	10%	15%	24%	100%	0.006
Ag85b	4	16%	32%	22%	30%	100%	0.014

Figure S5B. Circular Dichroism analysis of the Sbi-III-IV-Ag85b fusion protein.

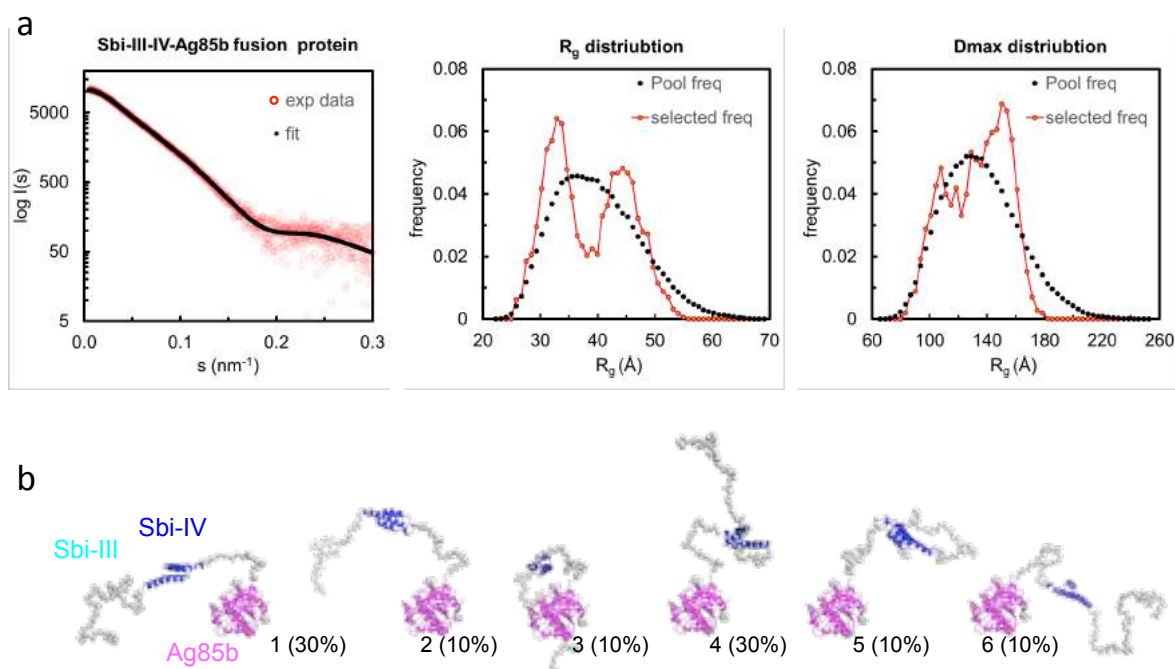

Figure S5C. SAXS analysis of the Sbi-III-IV-Ag85b fusion protein.

EOM modelling of the fusion protein Ag85b-Sbi-III-IV. a) Left panel, fit of the selected ensemble of conformers to the experimental scattering. Radius of gyration, R_g (middle panel), particle maximum dimension, Dmax (right panel), and distribution histograms of the selected conformers versus the pool. b) Examples of rigid body models of the selected conformers corresponding to the histogram peaks. The volume fraction of each species is indicated.

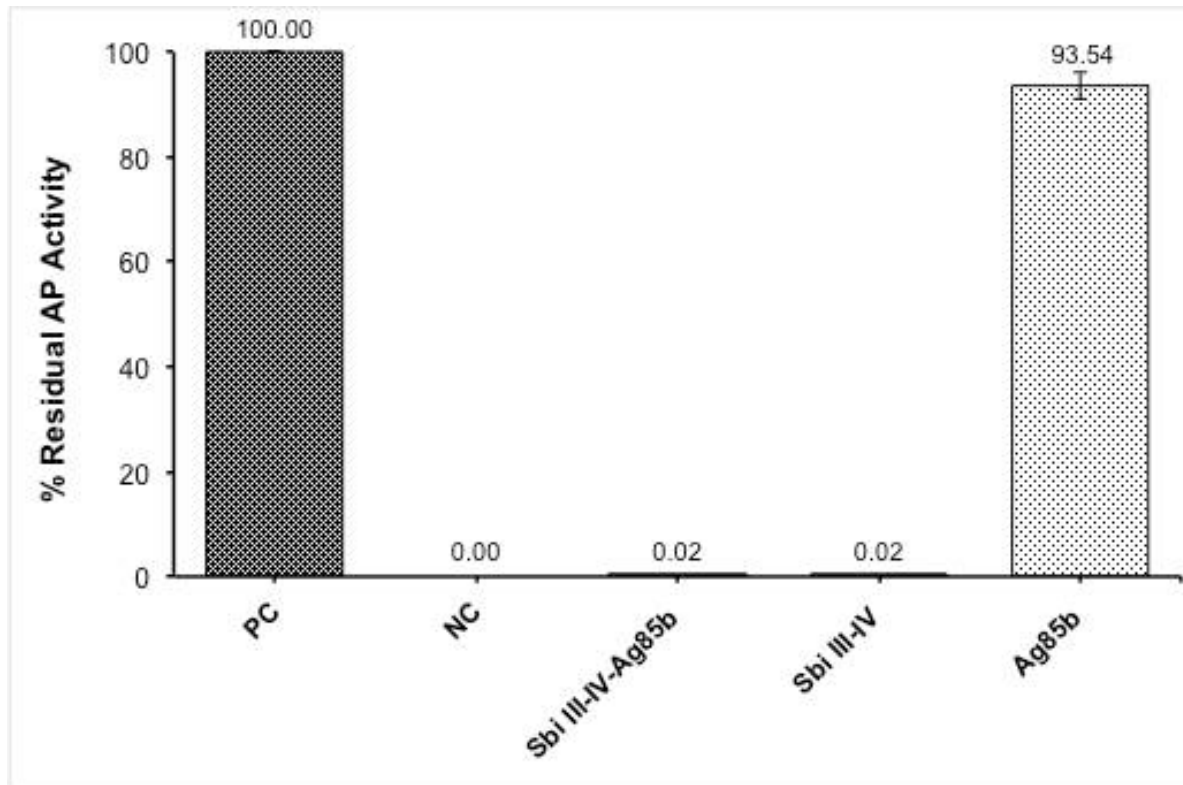

Figure S5D. Alternative Pathway activity analysis of the Sbi-III-IV-Ag85b fusion protein.

Analysis of Sbi-III-IV-Ag85b (4 μ M), Sbi-III-IV (4 μ M) and Ag85b (50 μ M) complement depletion activity using the Wieslab alternative pathway activity assay. Sbi III-IV-Ag85b displays strong complement depletion comparable to Sbi III-IV whereas an excess of Ag85b lacks this function and exhibits alternative pathway activity similar to the NHS positive control. Data are displayed as mean values from at least 2 replicates and error bars represent the standard deviation from the mean.

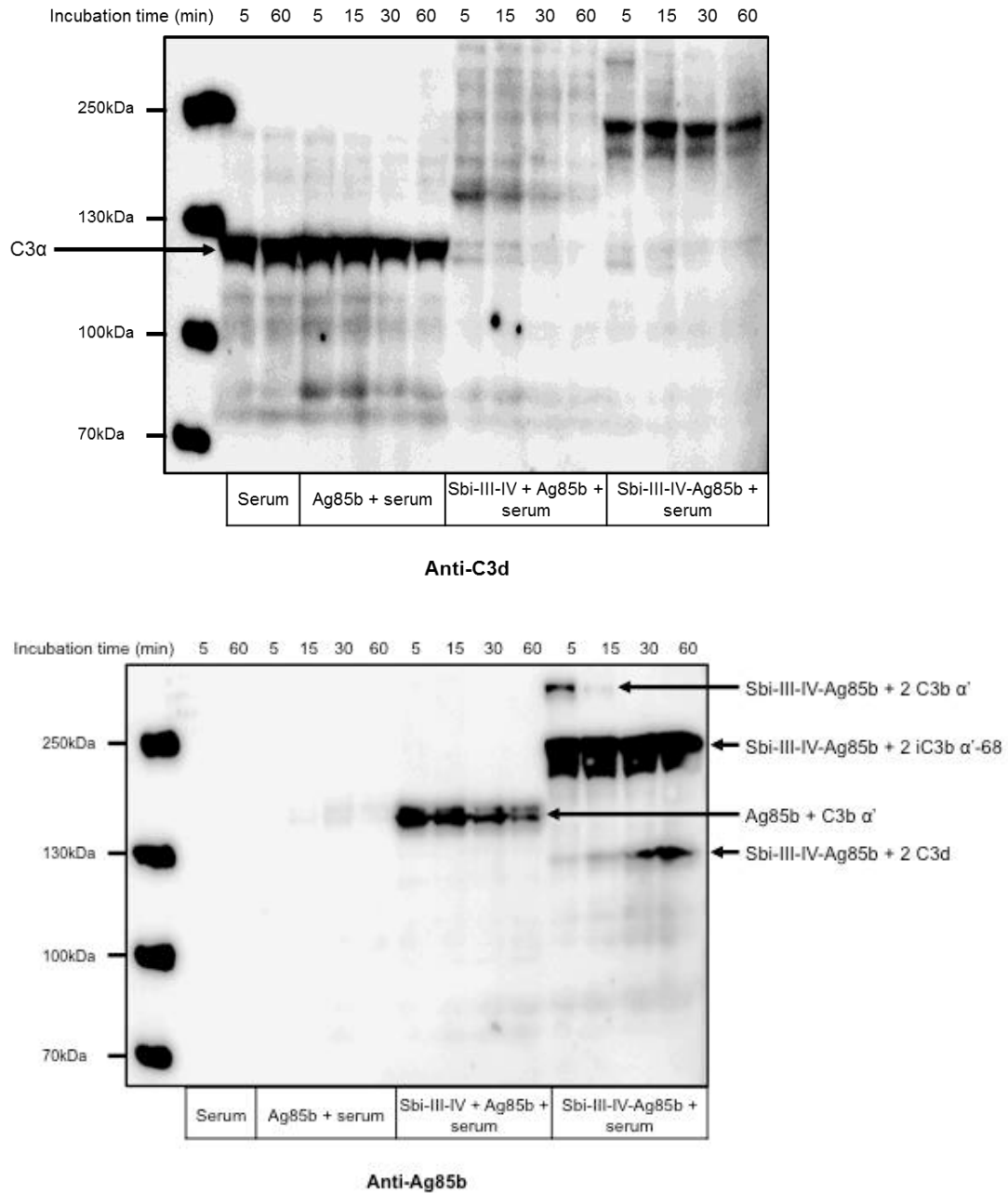

Figure S5E. Western blot analysis of C3 activation and C3-fragment deposition in NHS after incubation with Ag85b alone, Ag85b + Sbi-III-IV and Sbi-III-IV-Ag85b fusion construct.

C3 activation and C3-fragment deposition in NHS after incubation with Ag85b (100 μ M), Sbi-III-IV (35 μ M) and Ag85b (65 μ M), or Sbi-III-IV-Ag85b (100 μ M) visualized using anti-C3d (top) and anti-Ag85b (bottom) western blot analysis. Ag85b alone is unable to activate C3 as depicted by the intact C3 α -chain (top). However, in the presence of Sbi-III-IV and Ag85b, C3 is cleaved and Ag85b is opsonised by one C3b α' . The Sbi-III-IV-Ag85b fusion is opsonised with two C3b α' which are subsequently degraded to 2 iC3b α' -68 and 2 C3d over time.
